# Small-Pox

**Published:** 1862-11

**Authors:** 


### SMALL POX.

Just at this moment, when the characters and relations of ovine variola are affording the material for much speculation and thought, it will be particularly interesting to our readers to lay before them the subjoined documents recently submitted to us respecting the early history of vaccination as practiced by Jenner, and its relation to the variolous disease in animals.

The first is a narrative forwarded to us by Mr. Thomas Watts, of Frampton-on-Severn, Gloucestershire, and extracted from the manuscripts of his grandfather, Mr. John Webb, of Wick, Gloucestershire, dated September, 1799. It affords an additional evidence of the knowledge of the protective power of the cow-pox existing amongst the farming people of that valley before Jenner, of Berkeley, made it known to the world, and worked out the great results with which all are familiar.

### I.—NARRATIVE OF MR. JOHN WEBB.

"Some time in the month of May, 1792, having twenty-four children collected together at a house in Doynton for the purpose of being inoculated, and a Betty Bowman, then aged eighty, accidentally coming in, she was asked by another woman present whether she had ever had the small-pox; to which E. B. replied in the negative, asserting, with a considerable degree of confidence, that she was certain she never should, having in her younger days caught the cow-pox from a cow that was infected by a man in the small-pox. Such an opinion naturally induced me to desire from her a more particular account of the circumstance, when I was informed that when she was twenty-three or twenty-four years old she lived in the service of a farmer, on whose estate, at a distance from the farm-house or any other habitation, there was a small cottage, together with some cowsheds; that the cottage was let to a (probably one of his laborers), who dying in the small-pox betwixt Michaelmas and Christmas, the bed and bed-mat on which he had lain were thrown out into the sheds; that a cow belonging to their dairy, being, as she termed very chilly, frequently went into the cowshed, and had been observed to lie down on or near the bed and mat; that shortly after the same cow was seized with the cow-pox, and the whole dairy, consisting of nine cows, sickened one after the other, till at length the milk was so bad that it could not be used, and of course the cows were suffered to go dry, till which time she constantly assisted in milking them; that soon after she was seized with rigors and pain in her limbs, had a tumour form in the right leg and axilla, and that three pustules appeared on the hand near the thumb, from which there was a discharge for some time (she believed about nine days); that (as before mentioned) she neither prior nor subsequent to that period had the small-pox, though she had frequently visited persons ill in it, and once in particular lay on a bed on which a person had died in that disease, the bedclothes only being changed. She likewise observed, that two or three persons who had had the small-pox were frequently among the cows, but received no infection. She likewise informed me, that she knew a Mary Hathaway, who milked infected cows at one time, and was not infected by them, but that at another time she was; that she likewise never had the small-pox prior or subsequent to that period, though she resided several years in Bristol."

The second is an interesting record relating to Mr. Benjamin Jesty, described as having been the first known person who introduced cow-pox by inoculation, at a date twenty-two years prior to Jenner's first experiment. For this narrative we are indebted to Mr. Alfred Haviland, Surgeon to the Bridgwater Infirmary:

### II.—THE PROTO-MARTYR TO VACCINATION.

"As a matter of medical history, I send you the following facts, which may prove interesting to your readers.

"At the Rose and Crown Inn, Nether Stoway, co. Somerset, my attention was drawn, on the 21st of May last, to a photograph taken from a larger portrait of a good specimen of the fine old English yeoman, dressed in knee breeches, extensive double-breasted waist coat, and no small amount of broadcloth. He was represented sitting in an easy chair, under the shelter of some wide-spreading tree, with his stick and broad-brimmed hat in his left hand; his ample frame was surmounted by a remarkably good head, with a countenance which at once betokened firmness and superior intelligence.

"I have been thus particular in describing the portrait, for I am not quite certain whether the photograph was taken from a drawing, an engraving, or an oil-painting; if, however, the source was an engraving, in all probability there are some copies still extant, which the curious in such matters may think worth collecting.

"On the back of this photograph is a copy of the epitaph on our subject, as follows:

"Sacred to the memory of Benjamin Jesty, who departed this life on the 16th April, 1816, aged seventy-nine years. He was born at Yatminster, in this country; and was an upright, honest man,—*particularly noticed for having been the first person (known) who introduced cow-pox by inoculation; and who, from his great strength of mind, made an experiment from the cow on his wife and two sons in the year* 1774. (From the tomb in the churchyard at Yatminster, Dorset.)"

"I am informed by his relative, Mrs. William May (*nee* Jesty) that when the fact became known that he had vaccinated his wife and sons, his friends and neighbors, who hitherto had looked up to him with respect on account of his superior intelligence and honorable character, began to regard him as an inhuman brute, who could dare to practice experiments upon his family, the sequel of which would be, as they thought, their metamorphosis into horned beasts. Consequently the worthy farmer was hooted at, reviled, and pelted whenever he attended the markets in his neighborhood. He remained, however, undaunted, and never failed from this cause to attend to his duties; and the secret of this bold conduct may be traced in his determined chin and nose and firm lips. After living to see another enriched and immortalized for carrying out the same principles for which he had been stoned thirty years before, he died of apoplexy, like Jenner, in 1816. Jesty's experiment on his family was performed in 1774; and Jenner's on the 14th of May, 1796, just *twenty-two years later.*"

